# Psychological Dimensions and Their Inner Relationships of College Students’ Network Civilization

**DOI:** 10.3390/bs12120483

**Published:** 2022-11-28

**Authors:** Yuling Liu, Xiaorong Shen, Huaquan Mi

**Affiliations:** School of Marxism, University of Electronic Science and Technology of China, Chengdu 611731, China

**Keywords:** network civilization, psychological structure, college students, value identification, psychological perception

## Abstract

Network civilization is a product of the rapid development of the virtual world. This study aims to investigate the psychological structure of college students’ network civilization and to explore the role of value judgment and value identification between college students’ psychological perception and value selection. In this study, 1096 college students (511 men and 585 women) completed the anonymous questionnaire on network civilization. They completed the scales of psychological perception, value judgment, value identification, and value selection. The total scale and each subscale both had high Cronbach’s alphas (0.90–0.97), indicating good reliability. Results indicated the following: (1) Psychological perception and value selection are positively correlated. (2) Psychological perception improves college students’ value selection by enhancing their value judgment. (3) Psychological perception may positively affect college students’ value selection via value identification. (4) There is a chain-mediating role between psychological perception, value judgment, value identification, and value selection. These testimonies also contribute to and provide an empirical basis for guidance strategies for the cultivation of network civilization and moral education among college students.

## 1. Introduction

In the 21st century, humanity has entered a virtual world characterized by digitization, networking, interaction, and virtualization [1]. Network civilization has gradually become the new form of human social civilization development. Network civilization is the transformation of the “information wave” into virtual civilization, which is also defined as the virtual civilization in cyberspace [2]. As a new civilization, network civilization plays a vital role in social development, spiritual culture, and the need to study the civilization of the virtual world [3,4,5]. However, the contemporary study tends to focus on the identification with the values of network civilization, Internet literacy [6,7], practices [8], cognitive direction [9], and guidance [10]. These studies are primarily theoretical and lack empirical research. The study of network civilization, from perception through behavioral selection, has received little attention. College students, in particular, are the most active in cyberspace and the most frequent Internet users. Meanwhile, these studies disregard the fact that the cultivation of network civilization is a dynamic process that necessitates value transmission and supervision for developing subjects and progressively shapes value perception, identity, and selection. Nonetheless, most academics define the essence of network civilization as value identification, ignoring the selection of value behavior. Identification and behavioral selection are not synonymous. Understanding the worth of college students’ network civilization is necessary for the subjects to act on their behavior following value identification, which is the ultimate goal of network civilization.

Consequently, because of its theoretical and practical significance, the significance of network civilization merits additional consideration. The cultivation of network civilization is a significant indicator of college students’ network moral education and study of values and is also related to college students’ overall development and growth. The psychological structure of college students’ network civilization is currently unclear and should be researched further. The purpose of this study is to explore the psychological structure of college students’ network civilization and the mechanism of value identification and value judgment in psychological perception and value selection by analyzing college students’ perception, judgment, identification, and selection of network civilization.

## 2. Literature Review

All nations and civilizational systems in human society have undergone historical transitions from their various civilizational stages, stages such as agricultural civilization, industrial civilization, information civilization, and today’s network civilization [11,12]. This is an objective historical development trend and a subjective strategic choice for the growth of nations. Network civilization, as an emerging form and field of civilization development of human society under the conditions of the information network society, refers to the state of human society’s development and its beneficial outcomes after joining the information network society [13]. In the second section of the Internet era (2020–2070), which will be flooded with ubiquitous computers and lead to enormous social transformations, we refer to the existing civilization as the network civilization [12]. The study of network civilization is focused on scientific marvels and the social repercussions of technological advancement. In terms of historical history, network civilization is both the inheritance and development of conventional civilization and the new form of material civilization handed down by traditional civilization. In terms of spiritual values, network civilization refers to the stage of development and the necessities of people’s spiritual and cultural lives, which are advancing in step with the level of social development.

Understanding the meaning and purpose of network civilization is essential to defining the psychological structure of this society. The state of human social development known as “network civilization” is exemplified by people’s use of the Internet, their cultural practices on it, their spiritual beliefs, and the various spiritual products that they have produced by using the Internet’s unique medium and platform, as well as their network ideologies and moral principles [14]. Network civilization, as the accumulation, cohesiveness, and precipitation of sophisticated and positive online cultural achievements, has a significant impact on the alteration of the ideological dynamics, development of values, and enhancement of skill quality among college students [15]. Tsai and other experts believe that the Internet is rapidly becoming a fundamental aspect of global civilization because of its abundance of knowledge, ease, and entertainment. The Internet has altered how people work and play [16]. Some experts even predict that the Internet may usher in a decentralized and deconcentrated social civilization that mobilizes every node of human society. Most domestic and international researchers concur that the network has created a new form of human social civilization, that network civilization has a direct influence on the spiritual civilization of society, and that network civilization is a significant indicator of social development and progress [9,17,18].

The network world is conducive to the flourishing of the spiritual self [19]. When discussing network civilization, it is necessary to investigate how individuals might realize their affiliation with the cultural order and value systems [9] that define network civilization. Once people identify with these cultural order and value notions, they will become firmly ingrained in their culture and represent the core principles of network civilization [20]. According to the theory of planned behavior (TPB), attitudes, subjective norms, and perceived behavioral control are the three primary variables of behavioral intentions. The more positive the attitude, the stronger the behavioral intention, and vice versa [21]. The knowledge, attitude, and practice (KAP) model theory proposes that knowledge (perception) and beliefs influence practice (behavior) [22,23]. According to earlier studies, when college students enter the network society, they establish certain psychological perspectives. Attitude is the self-assessment of the phenomenon and behavior of network civilization. Subjective norms and perceptual behavioral control influence judgment and identification with network civilization, ultimately determining behavioral intention and influencing value selection. Based on KAP and TPB, we proposed the existence of mediating variables between psychological perception and the value selection of network civilization cultivation, i.e., value judgment and value identification.

Civilization in the cyber world can be considered as the recognition of cyber morality, and the final value selection of individuals can be regarded as the identification and selection of moral behavior. Moral identity theory proposes moral judgment and moral identity as significant measurement elements for the research into moral behavior [24]. Moral identity is another crucial concept of moral cognition derived from social identity theory. Typically, morality consists of three elements: behavioral standards, moral judgment, and moral action [25]. Because moral judgment does not inevitably lead to action [26], scholars have established the concept of moral identity. Moral identity is the foundation for social identification and is regarded as a self-regulatory system that motivates moral action [24]. Thinking about moral identity can be traced back to Blasi’s self-model [27]. It proposes a connection between moral judgment and moral action. Simply put, when a person’s identity is ethically focused, moral judgments may predict moral behavior more accurately when filtered through accountability judgments. In addition, social identity theory claims that a portion of a person’s self-concept derives from their view of their participation in a social group and their perception of the value or emotional significance of that membership [28]; this is the initial psychological perception. Moreover, recent empirical research indicates that moral judgment and identity shape moral behavior [29]. This indicates that the interaction between moral judgment and moral identity impacts moral behavior. Research based on moral behavior also found that moral judgment is the most critical factor influencing moral behavior, and moral identity influences moral behavior [29]. Therefore, the value selection of college students’ network civilization is the moral behavior in the network society. It is necessary to analyze the combined effect of value judgment and value identification on the value selection of network civilization based on college students’ psychological perceptions, when examining the psychological structure of network civilization.

In conclusion, this study is based on the psychological structure of the network civilization of college students. Using the knowledge, attitude, and practice model, perspectives of planned behavior theory, and moral identity theory, this study explores the psychological structure of college students’ network civilization cultivation, i.e., the interrelationship between psychological perception, value judgment, value identification, and value selection, a structure that is essential for understanding network civilization and promoting the development of network morality in virtual space.

## 3. Research Hypothesis

One must examine each connection between psychological perception and behavioral selection to investigate the psychological structure of college students’ network culture. The realization of the value of network civilization is the selection of behavior. This will also mark the success of human civilization in the 21st century [2]. The theory of planned behavior proposes that correct perceptual behavior directly predicts the likelihood of the behavior occurring and that behavioral beliefs induce either positive or negative attitudes toward behavior [30,31]. There is a connection between perception and behavior, as demonstrated by research showing that the perception of any social stimulus activates various associated knowledge in memory [32], social perception directly influences behavior, and perception and behavior are interconnected [33]. Meanwhile, the knowledge, attitude, and practice (KAP) model theory proposes a progressive relationship between knowledge (perception), beliefs, and behavior [34]. When humans enter the virtual world of network information, they form perceptions and memories of the network world in their brains, which is the starting point of the psychological structure of network civilization, i.e., psychological perception. Earlier research has indicated that from the standpoint of cognitive psychology, college students’ psychological understanding of values can promote individual and direct behaviors [35]. This form of psychological perception for network civilization is the initial value experience gained by the cultivation subject. This type of valuable experience has active implications, like an invisible hand searching for and capturing the attributes of objective objects to meet the subject’s needs. Once found, it effectively forms a psychological perception and a psychological value, thus introducing the subject to the value world from the fact world [36]. This indicates a connection between people’s perceptions of the network world and their value selections. Based on the available research, we propose the first hypothesis:

**H1:**  *Psychological perception significantly and positively influences value selection*.

Moral development theory states that moral judgment is a necessary but insufficient condition for moral action [37], and moral judgments are more-accurate predictors of actual moral behavior [38]. Related studies also confirm that moral judgment and moral action are congruent [39,40]. Various research has demonstrated that value judgment enters the psychological cognition process [41] and elevates psychological perception to a more reasonable level [42]. The value judgment determines whether something is meaningful, how meaningful it is, and whether its presence is necessary [43]. Human society is interactive in terms of the intrinsic prescripts of value judgment, and the social properties of humans mandate that they cannot renounce value judgment and engage in social collaboration [44]. Consequently, value judgment is a continuation of psychological cognition and determines the final direction of value selection. On the basis of the above literature, we propose the following hypotheses:

**H2:**  *Psychological perception is positively associated with value judgments*.

**H3:**  *Value judgment mediates the relationship between psychological perception and value selection*.

According to other researchers, moral judgments shape moral behavior [45], and moral decision-making begins with a perception of the moral issue [46]. In further research on moral behavior, studies have confirmed that moral identity is also a key determinant of moral behavior and that the interaction between moral judgment and moral identity influences moral behavior [29]. Moral identity helps address the judgment–action gap and has become an essential component of the theory of moral development [26,47]. In addition, Blasi showed that moral judgments more reliably predict moral behavior [27], and moral identity is the unity of moral and ego systems [48]. Value selection entails weighing and selecting among “many” and is a decision made by the subject following a rational examination and comparison of numerous value relations [42]. This demonstrates that value selection has more-evident value pursuit and valuable purpose than value identification and is the value subject’s active and voluntary conduct. Previous research has demonstrated that values and human behavior are interdependent and that a person’s adoption of culture results in the acquisition of a particular manner of behaving and the establishment of particular values [49]. The psychological structure of college students’ network civilization must progress from perception, judgment, and identification to forming a value selection. Values give a scale for determining the value relationship between people and the world, guide individuals’ practical and cognitive activities, and determine the value direction of those actions [50]. Value selection is a choice and practice of moral behavior, a logical corollary of psychological perception, judgment, and identification with values. Therefore, when exploring the network civilization of college students on the basis of the above literature, it is vital to comprehensively analyze the influence of moral judgment and moral identity on conduct, and from this analysis, we propose the following hypotheses:

**H4:**  *Psychological perception is positively correlated with value identification*.

**H5:**  *Value judgment is positively correlated with value identification*.

**H6:**  *Value identification mediates the relationship between psychological perception and value selection*.

**H7:**  *Value judgment is positively correlated with value selection*.

**H8:**  *Value identification is positively correlated with value selection*.

**H9:**  *Psychological perception influences value selection via the mediation chain of value judgment and value identification*.

## 4. Materials and Methods

### 4.1. Data Source

In this study, 1200 individuals from 30 institutions across China were utilized as study subjects, and finally, 1096 valid questionnaires were obtained (from 511 men and 585 women), with a valid return rate of 91.33%. Of these, 156 were first-year undergraduates, 292 were second-year undergraduates, 218 were third-year undergraduates, 147 were fourth-year undergraduates, 246 were in the master’s program, and 37 were in the doctoral program. We created and disseminated the study utilizing an online survey method (www.wjx.com (accessed on 20 July 2022). All respondents consented to this study when opening the survey link, and their participation was entirely voluntary and uncompensated. The responses of the participants were kept private. Overall, the survey subjects represent gender, grade, and major. Refer to Table 1.

### 4.2. Research Design

#### 4.2.1. Questionnaire Compilation

This study, based on the mature scales and existing literature studies, conducted a prestudy of college students from different disciplinary backgrounds through open-ended questionnaires and interviews and analyzed the variables that emerged from them, and the study’s dimensional indicators and items contents were interpreted and supported by experts in relevant research domains. According to the eight-part guidelines of the Opinions on Strengthening the Construction of Cyber Civilization released by the Chinese government website in September 2021, the general requirements, ideological leadership in cyberspace, cultural cultivation in cyberspace, moral construction in cyberspace, code of conduct in cyberspace, ecological governance in cyberspace, cultural construction in cyberspace, and organization and implementation are combined with college students’ online behavior and ideological and political education, together constituting the “Presurvey Questionnaire on the Cultivation of Network Civilization among College Students”. A 5-point Likert scale was used to score the survey. Indicators range from 1, for strongly disagree, to 5, for strongly agree, where 4 is for somewhat agree, 3 is for not sure, and 2 is for somewhat disagree. In this study, 120 college students from the corresponding author’s university were chosen for pretesting, and the data were subjected to a *t*-test, correlation analysis, and regression analysis using SPSS 26.0. The questions were subsequently deleted according to the structural validity coefficients of the four subscales, primarily the factor analysis loadings. The questions with ambiguous meanings and generalized questions were specifically mothballed. The scale’s content validity was strengthened further through peer-reviewed evaluation and testing. Lastly, the questionnaire was separated into two sections: (1) respondent demographics and (2) psychological perception, value judgment, value identification, and value selection subscales (See Supplementary Materials).

#### 4.2.2. Reliability and Validity Test

This research started by conducting KMO and Bartlett tests on the full scale comprised of psychological perception, value judgment, value identification, and value selection. The KMO and Bartlett tests were statistical studies used to validate the data’s applicability as exploratory factors [51]. A KMO value of over 0.90 is optimal [52], and the values of the Bartlett test indicate that the null hypothesis must be rejected when there is a significant level of 0.05 [53]. The results of this scale showed: KMO = 0.964, and for the Bartlett test, χ^2^ = 31,381.406, df = 465 (*p* = 0.000), which indicates that the sample data was appropriate for factor analysis. In this study, variables were extracted by using principal component analysis with varying–maximizing orthogonal rotation and extraction criteria of eigenvalues greater than 1. The rotational axis method is the maximum variance method of the orthogonal rotational axis method with a minimum factor of 0.5, which is banned, and sets a fixed number of extraction factors. There are 15 psychological perception measures, 16 value judgment measures, 21 value identification measures, and 15 value selection measures. Items with factor loadings less than 0.5 were eliminated following EFA. The scale retrieved four common components that explained 73.600% of the variation (more than 60%). The four factors recovered were psychological perception, value judgment, value identification, and value selection, as anticipated by the study. The analysis’s findings demonstrated that it reflected in the reasonable design of the scale dimensions, and the loading values of each indicator question item on their respective factors were greater than 0.6, indicating that the scale had good construct validity. There are seven measurement items for psychological perception, eight measurement items for value judgment, nine measurement items for value identification, and seven measurement items for value selection. The particulars are included in Table 2.

The reliability test revealed that the internal consistency, reliability, and stability of the scale were all satisfactory. According to Table 3, the Cronbach’s coefficients for the psychological perception scale are 0.932, the value judgment scale is 0.950, the value identification scale is 0.942, and the value selection scale is 0.951. Each variable scale obtained a Cronbach’s alpha coefficient of over 0.7, indicating a high degree of dependability (Table 3).

In this study, Amos24.0 confirmatory factor analysis (CFA) was conducted on the results of exploratory factor analysis (EFA) to verify the efficacy and stability of the factor structure, and the combined reliability was used to measure the reliability quality of each dimension. Composition reliability (CR) and the average variance extraction (AVE) were adopted to evaluate convergent validity [54]. All indices are more than or equal to 0.5, indicating that the model has outstanding convergent validity. The value of the square root of AVE is greater than the value of the correlation coefficient, indicating that the constructs have discriminant validity [55]. According to Table 3, the factor coefficients of psychological perception, value judgment, value identification, and value selection, corresponding to each question item, are all greater than 0.5, indicating that each latent variable corresponding to the question item to which it belongs is representative. Additionally, the average variance extractions (AVEs) of psychological perception, value judgment, value identification, and all reliability measures for value selection are better than 0.5, and the overall reliability (CR) is greater than 0.8, which shows that the model has reasonable reliability and validity.

**Figure 1:**
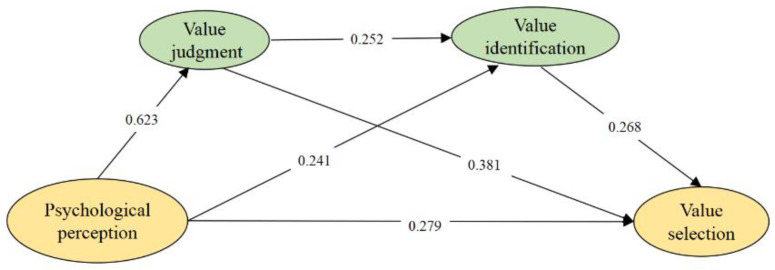
Theoretical model.

According to Table 4, the following are the fit index values: the value of χ^2^/df is 4.989, which is less than 5, and the fit is excellent; the RMSEA is 0.060, which is less than 0.08, and the fit is excellent. GFI has a value of 0.89, which is greater than 0.8, indicating a perfect fit; IFI has a value of 0.945, which is greater than 0.9, indicating a perfect fit; and CFI is 0.945, which is greater 0.9, and thus, the fit is acceptable. In conclusion, all correlation values fall within the allowed range [56,57], i.e., the overall model fit is satisfactory. Hence the current sample size of 1096 is adequate to generate statistically significant test results (as shown in Table 4 and Figure 1).

According to Table 5, there were significant correlations (*p* < 0.01) among psychological perception, value judgment, value identity, and value selection. Additionally, the latent variables have strong discriminant validity, as seen in the correlation coefficients between them being all lower than the square root of AVE. There are definite correlations and levels of difference between each latent variable.

## 5. Results

### 5.1. Theoretical Model

The four measuring dimensions are standardized: psychological perception, value judgment, value identification, and value selection. To evaluate the direct impact of psychological perception on value selection, model 1 is first created using psychological perception as the independent variable and value selection as the dependent variable. Second, to build model 2 and look at its parallel mediating effects, value judgment and value perception are included as independent mediating factors. Third, a connection between value judgment and value identification is made using model 2; that is, the chain-mediating path of “psychological perception → value judgment → value identification → value selection” is established in model 3, which explores the chain-mediating roles of both. The model is shown in Figure 1.

According to Table 6, Hypotheses H1, H2, H4, H5, H7, and H8 are statistically significant, and their respective paths are confirmed by empirical data. Specifically, the standardized path coefficient of psychological perception on value judgment is 0.623 (*p* < 0.001), indicating that there is a significant positive influence of psychological perception on value judgment; the standardized path coefficient of psychological perception on value identification is 0.241 (*p* < 0.001), indicating that there is a significant positive influence of psychological perception on value identification. The standardized path coefficient of value judgment on value identification is 0.252 (*p* < 0.001), indicating that there is a significant positive influence of value judgment on value identification. The standardized path coefficient of psychological perception on value selection is 0.279 (*p* < 0.001), indicating that there is a significant positive influence of psychological perception on value selection. The standardized path coefficient of value judgment on value selection is 0.381 (*p* < 0.001), indicating that there is a significant positive influence of value judgment on value selection. The standardized path coefficient of value identification on value selection is 0.268 (*p* < 0.001), indicating that there is a significant positive influence of value identification on value selection. Therefore H1, H2, H4, H5, H7, and H8 are verified.

### 5.2. Mediating-Effect Analysis

To analyze the mediating effect, this study used the bootstrap method to test the significance of the mediating effect. In the original data, a random sample was repeated 2000 times, and 95% confidence intervals were calculated. According to Table 7, value judgment and value identification mediates the association between psychological perception and value selection, and the mediating effect is statistically significant. The total mediating effect is 0.345, and 95% CI is [0.292, 0.408], accounting for 55.29% of the total effect of psychological perception on value selection. The total mediating effect consists of three indirect effects. The first path is psychological perception → value judgment → value selection. The effective value is 0.238, and 95% CI is [0.185, 0.310], accounting for 38.14% of the total effect. The second path is psychological perception → value identification → value selection. The effective value is 0.065, and 95% CI is [0.037, 0.100], accounting for 10.42% of the total effect. The third path is psychological perception → value judgment → value identification → value selection. The effective value is 0.042, and 95% CI is [0.027, 0.067], accounting for 6.73% of the total effect. The CI score of all mediating effects does not contain 0, indicating that all mediating paths are established. The direct effect of psychological perception on value selection is 0.279, while the total effect is 0.624.

## 6. Discussion

This research seeks to understand the link between psychological perception and value selection and understand the mediating effects of value judgment and value identification between psychological perception and value selection. It also seeks to understand the psychological structure of college students’ network civilization cultivation. The study’s findings are listed below.

### 6.1. Psychological Perception Significantly and Positively Influences Value Selection 

This study discovered that value selection was significantly positively predicted by psychological perception. This is in line with theory 1. The direct influence of psychological perception on value selection, with a direct impact value of 0.279, can be seen from the data to account for 44.71% of the total effect. According to the findings, college students with a higher level of psychological perception about network civilization will also have a higher level of value selection; on the other hand, students with a lower level of psychological perception will have a lower level of value selection. The results validate the theoretical model of planned behavior and the KAP model, which predicts that perception can predict the occurrence of behavior [30,31]. This indicates that psychological perception positively influences value selection. This is consistent with the results of previous studies [22,35,58]. Once humans enter the virtual world, they form perceptions and memories of it [32]. Perception has a direct effect on behavior, and perceptions are interconnected [33]. When researching the network civilization of college students, we begin with their psychological perspective of the network environment, which influences their behavior. The results of this study also demonstrate that psychological perception is positively correlated with value selection (behavior selection), further demonstrating the significance of psychological perception in the cultivation of network civilization.

### 6.2. The Mediating Role of Value Judgment and Value Identification

This study also studies the mediating role of value judgment and value identification between psychological perception and value selection. Specifically, it examines the indirect relationship between psychological perception and college students’ value selection. Value judgment and value identification were discovered to be significant mediating variables in college students’ psychological perceptions and value selections. This is also consistent with the structure of morality, in that moral judgment is an intrinsic aspect of morality. In network society, value judgment is a component of the network civilization mechanism [25]. This also demonstrates that moral identity theory is equally applicable to network civilization; i.e., value identification plays a fundamental role in the selection of values and influences the selection of civilized behavior on the Internet. According to moral development theory, moral action is influenced by moral judgment and moral identity. After college students’ psychological impressions of network civility have been formed, judgment and identification can more accurately predict moral behavior [27,29,39,59].

First, value judgment mediates the relationship between psychological perception and value selection; hence, psychological perception can affect college students’ value judgment and value selection. Hypothesis 2 was confirmed. Moreover, with an impact size of 0.238, this path has the biggest proportion of the total indirect effect, and the mediating effect accounts for 38.14% of the total effect. The mediating effect is statistically significant and the most significant mediating variable. This is consistent with earlier studies indicating that value judgments depend on the subject’s subjective value orientation and that various components, including experience, logic, sensibility, and emotion, contribute to forming value judgments [41,44]. The establishment of Hypothesis 2 also reaffirms that moral judgments can predict moral behavior [38]. In the virtual world, the theory of moral development also applies, and value judgment in the network civilization plays an essential role in value selection.

Second, the results demonstrated that value identification independently mediates the relationship between psychological perceptions and value selections; i.e., psychological perceptions impact the value selections of college students via value identification. Hypothesis 3 was confirmed. This path’s effect size was significant, accounting for 10.42% of the overall indirect effect, and its effective value was 0.065. This suggests that the value identification of college students is a rational identification with values that operates through emotional identification and does so actively and voluntarily after psychological perception, which produces a clear perception of value selection; i.e., value identification is a type of shared values that people use to regulate their actions in their social practice activities, which is consistent with earlier research findings [60]. Similarly, this finding is consistent with another research in that it finds that value identification appears in the form of ideas but ultimately manifests itself through value-behavior activities; i.e., value identification has a mediating role, and value identification is not only the construction and logical expression of ideas but also the practical activity of value behavior, which can improve college students’ value selection and behavior practice. In conclusion, this research demonstrates the importance of value identification as a mediator between psychological perception and value selection.

### 6.3. The Chain-Mediating Roles of Value Judgment and Value Identification in the Influence of Psychological Perception on Value Selection

This study also indicates the chain-mediating roles of value judgment and value identification between psychological perceptions and value selection. This research’s primary theoretical premise, Hypothesis 4, has been examined. The value of the chain-mediating effect is 0.042, indicating that the psychological cognition of college students can influence value selection via the chain mediation of value judgment and value identification. This also provides possible explanations for how college students’ psychological perceptions influence value selection and how they behave in a civilized manner online after their psychological perceptions. Moral development theory states that moral behavior is a complex phenomenon that is influenced by multiple factors (e.g., moral judgment and moral identity) and exhibits differences [29]. From the existing theory and literature, we have concluded that moral judgment and moral identity are the determinants of moral behavior [31,32,46,48]. The research results also verified this conclusion that people’s value judgment and value identification play a chain-mediating role in psychological perception and value selection in network society. This result also provides a reasonable explanation for the highest-value pursuit cultivated by network civilization. That is, the realization of the value of college students’ network civilization requires the subjects to act on their behavior after value identification, which is the ultimate goal of network civilization [2].

The psychological structure of network civilization and the origin of value formation in college students’ network civilization are psychological perceptions, which are their direct perceptions of the object properties and value relationships that satisfy their demands and goals. In this process, value judgment will be formed in the subject’s psychological perception and understanding, raising the psychological perception to a more rational level. Value judgment is a necessary prerequisite for values to be formed; i.e., college students should be able to evaluate values to ensure the right and scientific selection of values. Value identity is based on psychological perception and value judgment, and the value point is accepted from the heart. Value selection has the ultimate value pursuit and value realization compared with value identification. Value selection implies that realistic behaviors are made after the cognition and identification of values. That is, behavioral identification is a logical corollary of cognitive identification, emotional identification, and value identification [61]. This research shows that value identification is founded on psychological perception and value judgment. Value selection is a valued behavior chosen when an individual identifies with a value and accepts its intrinsic points [42]. Using social cognitive theory and educational psychology to evaluate the psychological structure between psychological perception, value judgment, value identification, and value selection, the results of this study can, in general, contribute to the study of college students’ network civilization. Overall, the study analyzes the psychological structure of college students’ network civilization cultivation on the basis of the knowledge, attitude, and practice model; moral development theory; and social identity theory, which will also draw more attention to the study of network civilization and virtual social civilization.

### 6.4. Limitations and Future Research Directions

It is necessary to describe the constraints of this inquiry. In this study, only a sample of Chinese college students was tested, limiting the generalizability of the results. In the future, validation of the model will require a different sample of people from other countries. Second, this study explored the psychological structure between college students’ psychological perception and value selection regarding the mediating role of value judgments and identification. However, with the further development of the digital era, there are more external environmental factors that will affect college students’ value choices, such as the fragmented information on the Internet and the evaluation system of network civilization. Future research should consider more variables to obtain more-convincing results and provide more-reliable action guidelines for specific practices. At the same time, this paper reveals the mediating role of value judgment and value identification between psychological perception and value perception, but with the continuous development of value judgment and identification, network society faces the challenge of multiple complex factors, and in the future, we have to pay attention to the influence and role of the interaction between personal identity, thoughts, and the virtual world on the civilized behavior on the network.

## Figures and Tables

**Table 1:** Demographic information of the participants.

Demographic Variable		Sample		
		Number	Percentage (%)	Cumulative Percentage (%)
Gender	Male	511	46.62	46.62
	Female	585	53.38	100.00
Grade	Freshman	156	14.23	14.23
	Sophomore	292	26.64	40.88
	Junior	218	19.89	60.77
	Senior	147	13.41	74.18
	Master	246	22.45	96.62
	PhD	37	3.38	100.00
Major	Natural Sciences	504	45.99	45.99
	Social Sciences	299	27.28	73.27
	Economic and Management	106	9.67	82.94
	Arts and Sports	79	7.21	90.15
	Others	108	9.85	100.00
Total		1096	100.0	100.0

**Table 2:** The orthogonal rotation component matrix of the scale.

	Component			
	Psychological Perception (PP)	Value Judgment (VJ)	Value Identification (VF)	Value Selection (VS)
PP1	0.794			
PP2	0.798			
PP3	0.759			
PP4	0.830			
PP5	0.816			
PP8	0.696			
PP15	0.709			
VJ2		0.770		
VJ3		0.779		
VJ4		0.786		
VJ5		0.796		
VJ6		0.839		
VJ7		0.835		
VJ12		0.778		
VJ16		0.724		
VF6			0.804	
VF7			0.816	
VF11			0.832	
VF13			0.723	
VF14			0.800	
VF15			0.793	
VF16			0.861	
VF17			0.762	
VF18			0.759	
VS3				0.774
VS4				0.784
VS11				0.774
VS12				0.702
VS13				0.769
VS14				0.778
VS15				0.763

Extraction method: principal component analysis; rotation method: maximum variance method.

**Table 3:** Reliability and validity examination.

Variable	Item	UC	SE	Z-Value	*p*-Value	SC	Cronbach’s a	CR	AVE
Psychological Perception (PP)	PP1	1.000				0.804	0.932	0.936	0.676
	PP2	1.184	0.036	32.751	***	0.845			
	PP3	1.098	0.036	30.199	***	0.798			
	PP4	1.055	0.029	36.174	***	0.904			
	PP5	1.027	0.029	34.944	***	0.883			
	PP8	0.758	0.028	27.135	***	0.736			
	PP15	0.825	0.029	28.918	***	0.773			
Value Judgment (VJ)	VJ2	1.000				0.850	0.950	0.953	0.719
	VJ3	0.940	0.027	34.647	***	0.821			
	VJ4	1.014	0.028	36.469	***	0.846			
	VJ5	0.835	0.022	37.796	***	0.863			
	VJ6	0.872	0.021	40.792	***	0.899			
	VJ7	0.894	0.022	40.109	***	0.891			
	VJ12	0.716	0.021	34.543	***	0.820			
	VJ16	0.664	0.021	32.206	***	0.786			
Value Identification (VF)	VF6	1.000				0.804	0.942	0.943	0.649
	VF7	1.009	0.032	31.844	***	0.827			
	VF11	1.000	0.031	32.598	***	0.841			
	VF13	0.871	0.031	27.821	***	0.750			
	VF14	0.950	0.031	30.655	***	0.805			
	VF15	0.868	0.029	30.209	***	0.797			
	VF16	0.955	0.027	34.897	***	0.881			
	VF17	0.735	0.025	29.222	***	0.778			
	VF18	0.709	0.025	28.244	***	0.758			
Value Selection (VS)	VS3	1.000				0.851	0.951	0.952	0.739
	VS4	1.001	0.027	36.889	***	0.852			
	VS11	0.983	0.027	36.028	***	0.841			
	VS12	0.857	0.025	34.175	***	0.815			
	VS13	0.933	0.024	39.397	***	0.883			
	VS14	0.912	0.023	39.302	***	0.882			
	VS15	0.894	0.022	40.028	***	0.890			

UC, unstandardized coefficients; SE, standard error; SC, standardized coefficients. *** *p* < 0.001.

**Table 4:** Goodness of fit index of the structural model.

Fit Index	χ^2^/df	GFI	RMSEA	IFI	NFI	TLI	CFI
Acceptable range	<5	>0.8	<0.08	>0.9	>0.9	>0.9	>0.9
Measured value	4.989	0.890	0.060	0.945	0.933	0.941	0.945

**Table 5:** Distinct validity.

	Psychological Perception	Value Judgment	Value Identification	Value Selection
Psychological Perception	0.822			
Value Judgment	0.623 **	0.848		
Value Identification	0.398 **	0.402 **	0.806	
Value Selection	0.624 **	0.664 **	0.533 **	0.860

** *p* < 0.01. The diagonal is the square root of AVE.

**Table 6:** The test results of path relationship.

Hypothesis	Path	Unstandardized Coefficients	SE	CR	*p*	Standardized Coefficients	Hypothesis Test
H1	PP → VJ	1.055	0.056	18.937	***	0.623	Established
H2	PP → VF	0.362	0.059	6.167	***	0.241	Established
H4	VJ → VF	0.224	0.034	6.535	***	0.252	Established
H5	PP → VS	0.439	0.049	9.047	***	0.279	Established
H7	VJ → VS	0.354	0.029	12.348	***	0.381	Established
H8	VF → VS	0.281	0.027	10.409	***	0.268	Established

PP, psychological perception; VJ, value judgment; VF, value identification; VS, value selection. *** *p* < 0.001.

**Table 7:** Results of the mediating-effect test.

	Effect	BootSE	Bias-Corrected 95% CI		Percentile 95% CI		Effect Amount
			Lower	Upper	Lower	Upper	
Direct effect	0.279	0.041	0.195	0.360	0.198	0.362	44.71%
PP → VJ → VS	0.238	0.031	0.185	0.310	0.179	0.302	38.14%
PP → VF → VS	0.065	0.016	0.037	0.100	0.036	0.099	10.42%
PP → VJ → VF → VS	0.042	0.010	0.027	0.067	0.025	0.063	6.73%
Total mediating effect	0.345	0.029	0.292	0.408	0.287	0.401	55.29%
Total effect	0.624	0.032	0.559	0.683	0.559	0.683	-

## Data Availability

The data from this study are available from the corresponding author upon reasonable request.

## Supplementary Materials

The following supporting information can be downloaded at https://www.mdpi.com/article/10.3390/bs12120483/s1, File S1: The main items of the questionnaire.
