# Supplementary material for: Psychological Dimensions and Their Inner Relationships of College Students’ Network Civilization
Source: Behav Sci (Basel). 2022 Nov 28;12(12):483. doi: 10.3390/bs12120483 (PMC9774907; doi:10.3390/bs12120483)
Supplement: Supplementary file 1 [file behavsci-12-00483-s001.zip › behavsci-2007854-supplementary.pdf]

Supplementary Material

The main items of the questionnaire of Psychological Dimensions and Their Inner Relationships of College Students' Network Civilization

Part I: Basic Information

1. Your gender is? ( )

A. Male B. Female

2. What is your grade? ( )

A. Freshman B. Sophomore C. Junior D. Senior E. Master's degree in progress F. Doctoral degree in progress

3. What is your major? ( )

A. Natural Sciences (Science, Agriculture and Medicine) B. Social Sciences (Literature and History) C. Economics and Management D. Art and Sports E. Other

4. Your family location belongs to? ( )

A.Town B.Rural

5. What is your school type? ( )

A. Ministerial college B. Provincial undergraduate college C. Private college D. High school E. Independent college

6. What is your age? ( )

A. After 90 B. After 95 C. After 00

7 Your academic performance in the class is? ( )

A. Top 20% B. Upper middle 20%-50% C. Lower middle 50%-75% D. Bottom 20%

8. The nature of your father's occupation is? ( )

A. Institutional personnel B. Professional and technical personnel (such as teachers, doctors, managers or practitioners, private business owners) C. Agriculture, forestry, animal husbandry and fishery D. Workers (manufacturing) E. Jobless/unemployed/layoff F. Retired

9. The nature of your mother's occupation is? ( )

A. Institutional personnel B. Professional and technical personnel (such as teachers, doctors, managers or practitioners, private business owners) C. Agriculture, forestry, animal husbandry and fishery D. Workers (manufacturing) E. Jobless/unemployed/layoff F. Retired

10. The time you came into contact with the Internet was? ( )

A. Primary school B. Middle school C. High school D. University

11. The length of your daily Internet access is? ( )

A. Less than 1 hour B. 1-3 hours C. 3-6 hours D. 6-9 hours E. More than 9 hours

12. The client you use most frequently to access the Internet is? ( )

A. Smartphone B. Tablet PC (such as iPad, etc.) C. Computer (PC terminal)

13. The main purpose of your Internet access is (up to 3 items)? ( )

A. Focus on national current affairs B. Focus on social hotspots C. Entertainment and leisure D. Social interaction E. Learning F. Online shopping G. Watching movies and TV programs/short videos H. Playing games I. Other ______

14. The social software you use most frequently is.? ( )

A. WeChat B. QQ C. Weibo D. TIKTOK E. Xiaohongshu F. Other ______

15. When you need to search for study-related materials and information, you usually use? ( )

A. Baidu, Google and other search engines B. Professional academic websites or journal databases C. Forum communities, posting bars and other online communities to inquire or seek help D. Sina Weibo, Zhihu E. Other ______

16. Does your school offer courses related to the cultivation of Internet civility among college students? ( )

A. Yes, B. No, C. Don't know

Part 2

Please read the following views or questions carefully and tick "√" on the corresponding options according to your true feelings about whether you agree or not.

		Strongly agree	Somewhat agree	Not sure	Somewhat disagree	Strongly disagree
**1. Psychological perception**						
PP1	You clearly our country network civilization construction related content.					
PP2	You understand the importance of the construction of network civilization to the development of national undertakings and people's lives.					
PP3	You understand the laws and regulations related to the construction of network civilization.					
PP4	You know exactly what you value.					
PP5	You understand the community of destiny in cyberspace.					
PP8	You understand your spiritual and cultural needs in cyberspace.					
PP15	You know about the platforms and major initiatives to cultivate and build network civilization (e.g. The "Qinglang" initiative).					
**2. Value judgment**						
VJ2	You think the cultivation of network civilization is a mandatory course for college students.					
VJ3	You think that false information on the Internet and cyber crimes have a negative impact on the moral quality of college students on the Internet.					

VJ4	Do you think the cultivation of network civilization will affect the development of society's economy, culture and education?					
VJ5	Do you think cyber-bullying and cyber-violence will have an impact on college students' network civilization literacy?					
VJ6	You think the need of college students for online spiritual culture influences the cultivation of network civilization.					
VJ7	You think the inheritance of Chinese Excellent Traditional Culture has an important influence on network civilization.					
VJ12	You think schools have an important role in cultivating network civilization among college students.					
VJ16	Do you think the products, activities and platforms of network civilization construction will influence the cultivation of network civilization?					
**3. Value identification**						
VF6	You agree to make the cultivation of network civilization an important part of your study and life.					
VF7	You agree that building a community of destiny in cyberspace will be the core of the future Internet global governance system.					
VF11	You think it is necessary to strengthen the inheritance of the Chinese Excellent Traditional Culture to improve the network civilization of college students.					
VF13	You agree with the importance of the school's efforts to improve college students' network literacy.					
VF14	You agree with the importance of family in improving college					

	students' network literacy.					
VF15	You think we need to strengthen the values and moral education of college students to improve the quality of network civilization.					
VF16	You think it is necessary to strengthen the fight against cyberbullying, cyber-violence and other harmful behaviors					
VF17	You think it is necessary to establish and improve the laws and regulations to combat cyber crimes.					
VF18	You think we should strengthen the protection of personal information, privacy and other data of Internet users.					
**4. Value selection**						
VS3	You will take the initiative to improve the quality of personal network ideology and morality.					
VS4	You will take the initiative to study and inherit the Chinese Excellent Traditional Culture to improve the quality of network civilization.					
VS11	You will take the initiative to learn and spread positive online cultural products and values.					
VS12	You will actively support and publicize, network civilization building activities					
VS13	You will take the initiative to strengthen the cultivation of network civilization in your daily life and study.					
VS14	You will take the initiative to learn and comply with the laws and regulations related to network civilization and be a good Internet user.					
VS 15	You will take the initiative to learn the protection of personal privacy information and data security protection methods on the Internet.					
